# Notes of Hospital Clinics

**Published:** 1897-08-21

**Authors:** 


### Medical Progress and Hospital Clinics.

[*The Editor will be glad to receive offers of co-operation and contributions from members of the profession. All letters should be addressed to* The Editor, at the Office, 28 & 29, Southampton Street, Strand, London, W.C.]

### Notes of Hospital Clinics.

[*Reported for* The Hospital, *and corrected by the authors.*]

### GUY'S HOSPITAL.

Clinical Lecture on a Case of Aneurism.

By L. A. Dunn, M.S., F.R.C.S., Assistant Surgeon to the Hospital.

The case is that of a man aged 39, an insurance agent, who was admitted on July 12th, 1897, for a large rapidly-growing pulsating tumour on the inner aspect of the right thigh and knee. The tumour was tense and discoloured; a loud bruit was heard at one part, a thrill was felt by the hand placed on it; there was no expansile distension, there was no pulse in the arteries below the tumour, and there was no œdema of the leg.

Now this tumour was one of two things—either a pulsating sarcoma of the femur, or an aneurism which had given way.

The patient's previous history was that he served 21 years in the army, and most of that time in India and Burmah. He had fever two or three times, and dysentery frequently. The last attack of fever was in 1884. He has had a syphilitic sore, and has also suffered from tonsillitis. He left the army in 1892, and since that time has done a great deal of walking daily in the pursuit of his employment. The history of the present illness is that 18 months ago he received a severe blow with a cricket ball on the inner and front aspect of the right thigh. The part immediately became greatly swollen, but the swelling subsided very rapidly. The skin remained discoloured for a month or six weeks.

On July 1st, 1897—*i.e.*, ten days ago—he first noticed pain and swelling of the part concerned, but could not account for it by a fall, or stumble, or anything of the kind. The swelling and inability to move the knee-joint increased rapidly, and on Saturday last, July 10th, I saw him an out-patient and admitted him.

The condition on admission was that there was a large swelling, oval in shape, which extended all along the inner side of the thigh above, but below it involved the knee and extended below it. Its greatest length from above downwards was eight inches. The whole direction of the swelling was slanting from above downwards and outwards. The circumferential measurement of the swelling was twenty-two inches; its greatest width was three and a-half inches. The swelling was not particularly well defined, nor were its edges markedly raised. It was very hard at the upper and inner part, but softer and rather fluctuating below. There was pain at the back of the swelling and along the upper part of its right border, but all the remainder was not tender to the touch.

I saw the case with Mr. Golding Bird, and later Mr. Bryant saw it. He came to the conclusion that it was a ruptured artery.

On July 16th the patient had been suffering great pain nearly all the previous night. All the measurements previously taken were found to be increased by an inch to an inch and a half.

On July 20th—the eighth day after admission—the tumour swelled rapidly and the skin was tense over it, and the patient suffered great pain. Two skiagraphs were taken to see if any spicules of bone were present in the tumour, or any roughening of the surface of the femur, such as might be expected if the tumour were a sarcoma, but nothing of the kind was seen. The temperature had been raised ever since admission, due probably to the tension.

It was now evident that if the tumour were not explored the leg would become gangrenous; so we put the patient under an anæsthetic and applied Esmarch's bandage round the upper part of the thigh by Jordan Lloyd's method. I then made an incision six inches in length along the whole length of Hunter's canal. Then I lifted the sartorius muscle on to one side, and incised the tumour. Immediately a lot of arterial blood spurted out, and then I removed handfuls of clot. Then I put my finger into the cavity and found the front of the femur denuded of periosteum. There was no pus. The femoral artery and vein were so matted together that it was almost impossible to separate them, so I put one ligature above at the top of Hunter's canal, and one below at the upper part of the popliteal space, and then removed the artery and vein between these ligatures. Several branches coming off from the sac had to be tied. Then I filled the cavity with boiled water, and wrapped the leg in cotton wool and elevated it. After the operation the patient was cold land col- apsed, but to-day he is comfortable and warm.

The denuded femur is a bad feature of this case, as the femur will necrose.

It is said that after removal or tying of the femoral artery and vein gangrene is produced; but as this patient had no pulse in the distal arteries for several days before the operation the collateral circulation was already doing the work; and, as the pressure on the vein was so great as to render it almost useless as a blood channel, the procedure seemed justifiable. In addition there was marked enlargement of the superficial veins, and the patient was getting collapsed, so that time was important. The case was, of course, an aneurism.

Now, about the femur being denuded of periosteum. The question is, Was it primary necrosis, or did the pressure of the aneurism destroy the periosteum? Well, we have all seen how an aneurism may destroy the periosteum covering a vertebra, and this may have been the case here.

This cavity was filled with boiled water after the operation. I consider this better than having it filled with germ-laden air, from which germs may be absorbed, or with boracic lotion from which the drug may be absorbed.

### SPRAINED ANKLE.

By E. Percy Paton, M.D., M.S., F.R.C.S., Surgical Registrar to Westminster Hospital.

A sprained ankle is one of the commonest accidents to the lower extremity, and though it may, if severe, be one of the most troublesome accidents to treat, rendering the limb useless for no inconsiderable period of time, it often receives very scanty attention, with the result that the trouble to which the lesion would in any case have given rise is considerably increased.

The question, What is a sprained ankle? may be answered in the following manner: It is any damage produced by a wrench or similar injury of the joint short of actual fracture or dislocation. Practically the condition found in a sprain varies very much. In a severe case various ligaments may be completely torn across, or, as these often prove even stronger than their bony attachments, a thin scale of bone may be torn off here. The exact ligament to give way depends upon the way the force has been applied, and as the accident is usually a twist of the foot either everting or inverting the sole, it is the deltoid or one of the three portions of the external lateral ligament which gives way. At the same time the synovial membrane may be widely torn and contused, while a considerable effusion, at first of blood and later of synovial fluid, takes place into the joint. In addition to the above, damage to the tendons and tendon sheaths around the articulation is often not inconsiderable. The tendons themselves are rarely torn, but their sheaths are often very considerably damaged—in some cases sufficiently to actually permit dislocation of the tendon—while at the same time extensive effusion into these sheaths, and also into the damaged soft parts around, is very common; and this blood, when it has spread to the skin, accounts for the extensive bruising, which makes its appearance in a day or two, and is usually most marked over the structures most injured, and so gives some indication of the direction in which the joint has been twisted, and therefore what structures have probably been torn.

In any case, where the damage has been anything like as serious as has just been described, it is easy to see that it may be a long time before the joint thus injured returns to a more or less normal condition, and these cases warrant the statement which has been made, that a badly sprained ankle is not infrequently a more troublesome injury than a simple fracture. If in addition to the injury the patient be old, in feeble general health, or especially of a gouty or rheumatic tendency, the tediousness of recovery may be still more marked. The changes which immediately follow the receipt of the injury are inflammatory in nature, resulting in a considerable effusion of serum and lymph into the joint, the adjacent tendon sheaths, and the surrounding tissues, thus much increasing the swelling, and later on, when the fluid part of the effusion has largely disappeared, leaving plastic lymph and blood clot, to some extent matting the parts together, and giving rise to considerable stiffness, making movement painful and difficult.

The *diagnosis* of a sprained ankle has to be made from a fracture or separated epiphysis. The latter usually presents no difficulty; it can only occur in the young (under the age of twenty; the lower epiphysis of the tibia uniting with the shaft at eighteen, that of the fibula at twenty). If it be the tibial epiphysis that is separated, considerable deformity will be present, the foot and ankle joint being displaced backwards; but being easily replacable with soft-rubbing crepitus. The accident is an exceedingly rare one. From fracture the diagnosis is made in a negative manner, by finding no excessive freedom of movement or crepitus, the latter being the important sign of fracture in cases where only a small piece of bone is broken off; it may, however, be completely concealed by the swelling and blood effused. A fracture low down in the shaft of the fibula is easily excluded by pressing the shaft of the fibula firmly towards the tibia at about its middle, when if the bone be intact it will be felt to distinctly spring under the hand. Should any doubt remain after careful examination, the case should be treated as a fracture until the swelling has subsided sufficiently to allow a satisfactory examination to be made, when the small displaced fragment of bone (*e.g.*, part of a malleolus) can usually be made out, especially as a little callus will probably by this time have been formed around its broken surface.

The *treatment* of sprained ankle is at first directed towards relieving pain, preventing increase of the swelling, and keeping down, as far as may be, the inflammation consequent on the injury. Most essential to these ends is perfect rest and elevation of the affected limb, while at the same time cold is applied either by means of an ice bag or Leiter's tubes or lint kept constantly wet with evaporating lotion (*e.g.*, lead lotion and spirit). The routine treatment at hospitals has commonly been to firmly bandage the joint with a wet calico bandage. Under these simple methods of treatment slight cases will usually rapidly get well, but care must be taken that the joint is not used until the swelling has subsided, the heat gone, and pain is practically absent. Not infrequently even a slight sprain will give rise to considerable subsequent pain and trouble for some length of time owing to its having been used too soon, when another couple of days' rest would have prevented this. Unfortunately, among the poorer classes, this day or two of rest is often impossible to get. In very severe cases more complete rest is found satisfactory, in order to obtain which a back splint with a foot piece, or what is better still, and often handier, a light plaster or starch bandage case may be applied. This may be put on at once, or a day or two may be allowed to elapse while cold is being used, and then the plaster may be put on. It should be put on with a certain amount of firmness, and in order to do this and prevent its being at the same time too tight a sheet of cotton-wool should be cut into strips and rolled into wool bandages, and with these the ankle should first be covered.

To make a starch case (this is sometimes more convenient than plaster, as starch is always easily obtainable, and the case when finished is lighter, though, of course not so stout), the ankle is covered with cotton-wool, as described above, and then bandaged with an absorbent water bandage, over each layer of which is painted some strong starch paste, and the result dried by the fire. When dry this usually forms a splint which is quite sufficiently rigid for the object in view. It has been advised by some to use an elastic (Martin's) bandage, also applied over cotton-wool; but this elastic pressure usually gives too much pain to be borne for long, and it is doubtful if the discomfort to which it gives rise is counterbalanced by any very great advantages. Sometimes the pain of a sprain is very considerable, and is quite unrelieved by any of the methods of treatment detailed above. Relief may then be sometimes given by a local hypodermic of morphia, or the application of two or three leeches over the most painful part, or by removal of the excessive fluid effusion by aseptic aspiration. The last method, however, is often not very satisfactory, as it is commonly the extra-articular effusion and extravasation, which can scarcely be drawn off, which gives rise to the pain by pressing on the nerves; while, if it be that within the joint, the fluid is often thickened by blood clot, and so does not run easily through an ordinary-sized aspirating needle.

So soon as the acute symptoms about the joint have gone, massage with passive movement must be commenced. In the slighter cases it may be done almost from the very first, while in the worst cases it may be carried out with care so soon as it can be borne, which is usually within the first week or so. The great local indication is the disappearance of excessive heat from the joint.

By carrying out friction in the direction of the circulation, it is often very striking how soon the extravasated blood and effusion disappears, and the parts return to their normal condition. This effect is produced partly apparently by mechanically moving on the plastic material, and so freeing the smaller vessels from local pressure, thus promoting absorption by earlier establishing a free circulation, while at the same time prolonged stiffness is prevented by the passive movement.

In some cases, however, even when carefully treated, considerable weakness and painfulness of the joint remains for some considerable period. If at the same time some limitation of movement also persists, this may very likely be due to some adhesions in the joint itself or in the tendon sheaths about it, when the best thing to do is to give the patient gas, and then freely move the joint, when a cracking sound, followed by ability to move the joint freely, will indicate, and at the same time usually cure, the condition.

In other cases, chronicity of the case is dependent upon a gouty or osteo-arthritic tendency shown by other signs of these troubles elsewhere, which must then be appropriately treated. In any case, indeed, which shows a tendency to hang fire, constitutional means for improving the general health must not be forgotten; the best of these, and one which often produces most beneficial effects in an otherwise intractable case, is a change of air and scene to the country or seaside.

